# Corrigendum: Autoantibodies in renal diseases – clinical significance and recent developments in serological detection

**DOI:** 10.3389/fimmu.2020.00424

**Published:** 2020-03-11

**Authors:** Gianna Mastroianni-Kirsztajn, Nora Hornig, Wolfgang Schlumberger

**Affiliations:** ^1^Division of Nephrology, Department of Medicine, Federal University of São Paulo, São Paulo, Brazil; ^2^EUROIMMUN Medizinische Labordiagnostika AG, Institute for Experimental Immunology, Lübeck, Germany

**Keywords:** autoantibodies, renal autoimmune diseases, anti-PLA2R, anti-THSD7A, anti-nucleosomes, anti-dsDNA, ANCA, anti-PR3

In the original article, there was a mistake in Figure 1 as published. The images of MPO and GBM microdots had been processed and display too high similarities.

**Figure 1:**
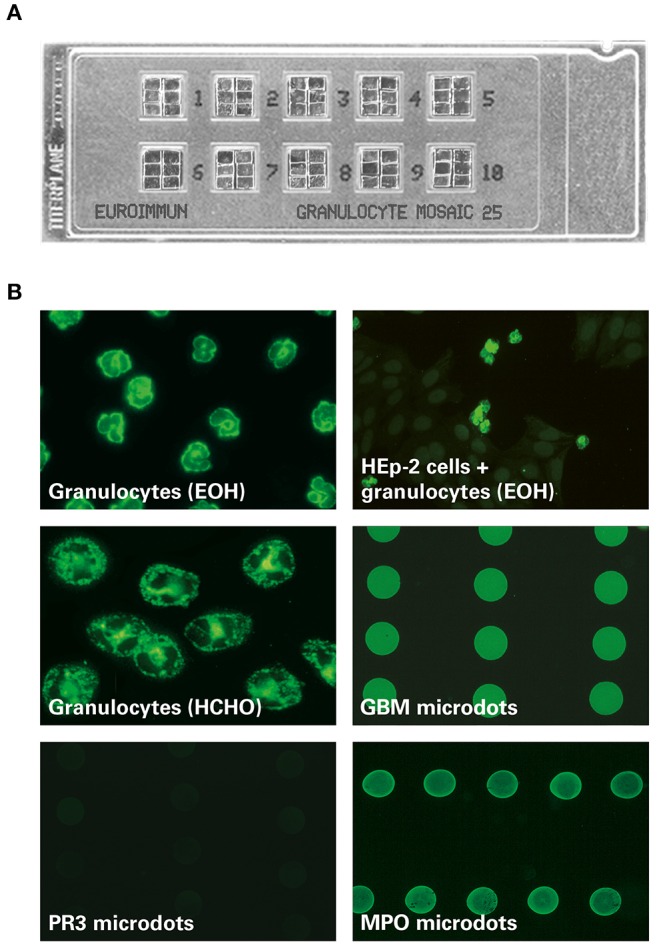
**MPO-ANCA fluorescence pattern on the EUROIMMUN EUROPLUS™ Granulocyte Mosaic (IgG)**. **(A)** Examplary microscope slide with 10 reaction fields, each containing 6 biochips forming a mosaic. **(B)** In the EUROPLUS™ Granulocyte Mosaic, each biochip represents a different substrate: ethanol-fixed granulocytes [granulocytes (EOH)], formalin-fixed granulocytes [granulocytes (HCHO)], HEp-2 cells in combination with ethanol-fixed granulocytes [HEp-2 cells + granulocytes (EOH)] as well as PR3, MPO, and GBM microdots. The exemplary images illustrate the reactivity of a patient sample positive for anti-MPO and anti-GBM: Besides a P-ANCA pattern on ethanol-fixed granulocytes and a granular C-ANCA pattern on formalin-fixed granulocytes, MPO-ANCA is characterized by a positive fluorescence signal on MPO but not on PR3 microdots. In addition a positive fluorescence signal on GBM microdots is shown.

In addition, the legend for Figure 1 was misleading. It has to be stated more clearly that the images are shown for illustrative purposes only. The correct Figure 1 and its legend appears below.

The authors apologize for this error and state that this does not change the scientific conclusions of the article in any way. The original article has been updated.

